# Supplementary material for: Mechanical interactions among followers determine the emergence of leaders in migrating epithelial cell collectives
Source: Nat Commun. 2018 Aug 27;9:3469. doi: 10.1038/s41467-018-05927-6 (PMC6110746; doi:10.1038/s41467-018-05927-6)
Supplement: Supplementary file 1 — Supplementary Information [file 41467_2018_5927_MOESM1_ESM.pdf]

Supplementary Information

Mechanical interactions among followers determines the emergence of leaders in migrating epithelial cell collectives

Vishwakarma et al.

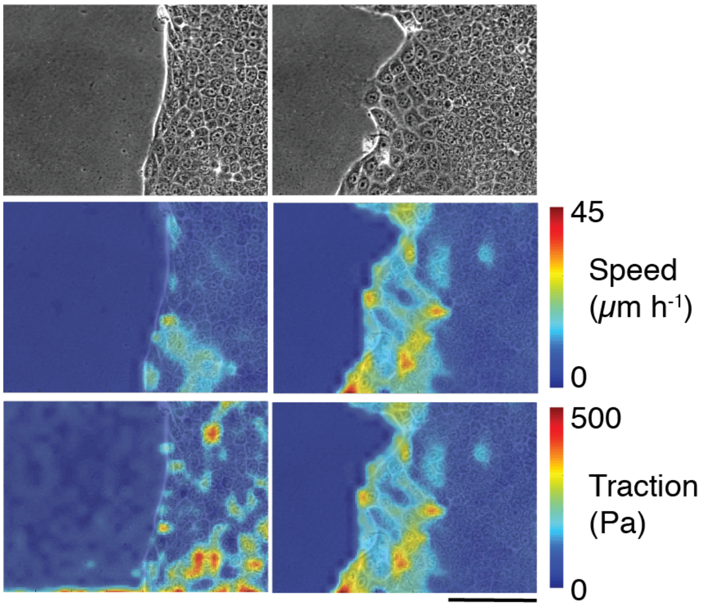

Supplementary Figure. 1. Physical activity of monolayer at different time points. Representative phase contrast images (*top*) immediately (*T = 0h, left panel*) and three hours *(T = 3h right panel*) after confinement release, corresponding speed (*middle*) and traction stress maps (*bottom*) showing very little or no migratory activity but adequate traction stresses in the followers immediately after confinement removal. Experimental repeats, n = 5. Scale bars, 100 μm.

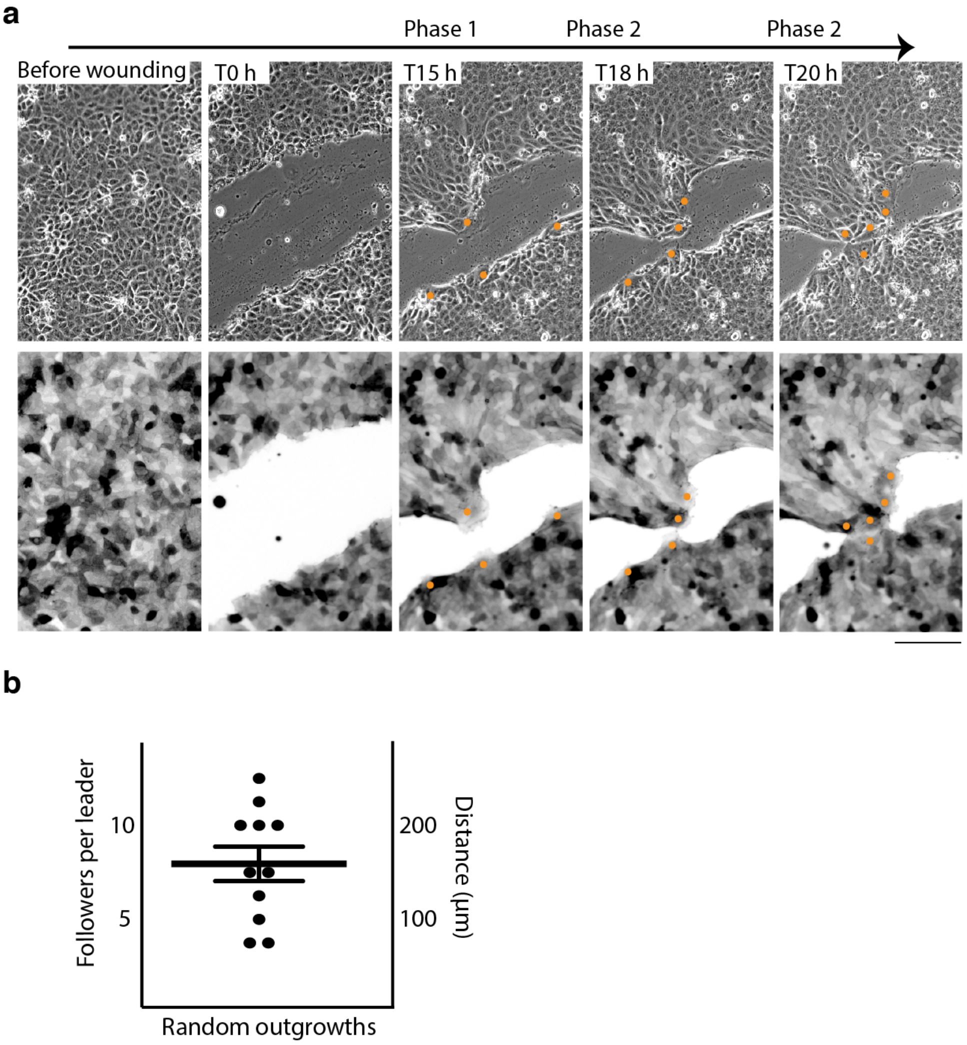

Supplementary Figure. 2. Scratch wound assay. (a) Representative phase contrast images (*top panel*) and actin images using *LifeAct* cells (*bottom panel*) showing biphasic migration of cellular outgrowth upon scratching a healthy monolayer. Leader cells are marked with an orange marker. **(b)** Scatter dot plot showing number of followers per leader in cellular outgrowths chosen at random time points after scratching showing number of followers per leader comparable to what was obtained using a confinement release assay (*figure 4*) **.** Right Y axis show cell-number converted to distance using mean cell diameter showing extent of followers comparable to force localization length, L_P (figure 4). Line represents mean and errors bars represents S.E.M. Data is pooled from three independent experiments, n = 3.

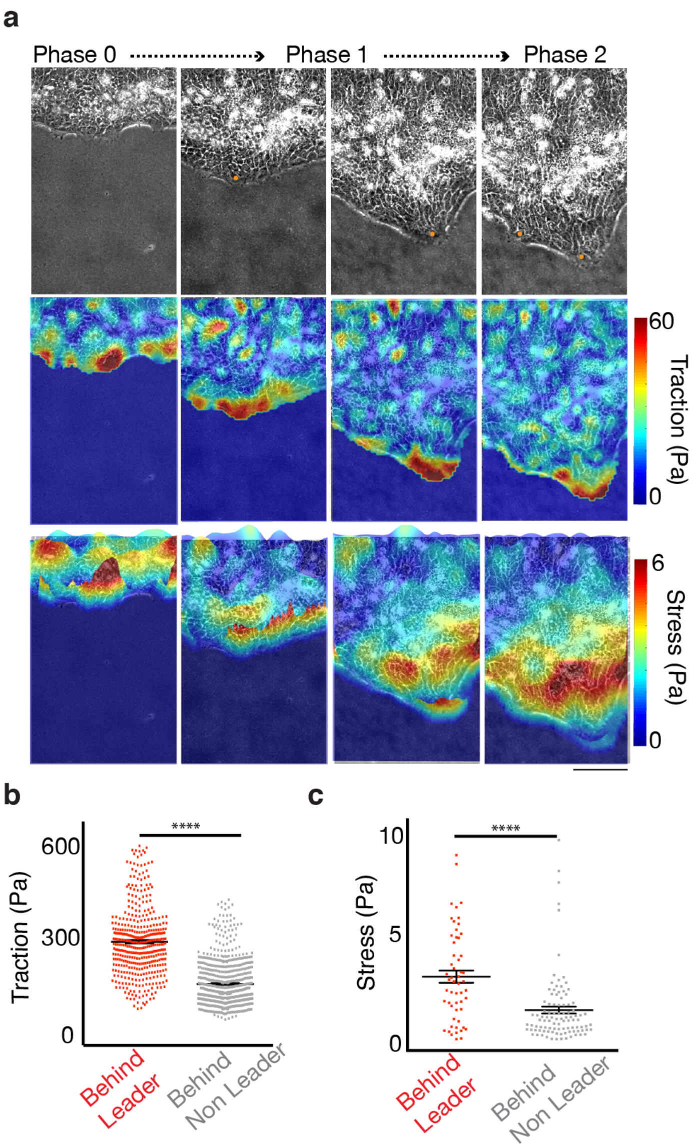

Supplementary Figure. 3: Force transmission from followers facilitate leader cell formation and regulate distance between leaders in HaCaT cells (a) Representative phase contrast images (*top*), corresponding traction force profiles (*middle*) and corresponding stress landscapes overlaid with phase contrast images (*bottom*), showing high forces in followers behind future leaders in different phases in HaCaT cells. Leader cells are marked with orange dots. **(b)** Scattered dot plot showing mean traction forces behind leader and non-leader cells in phase 0. **(c)** Scattered dot plot showing mean of average normal stress behind leader and non-leader cells in phase 0. Lines represent mean and error bars represent S.E.M. **** P<0.0001, Mann-Whitney test. Scale bar = 100 µm. Data is pooled from three independent experiments, n = 3.

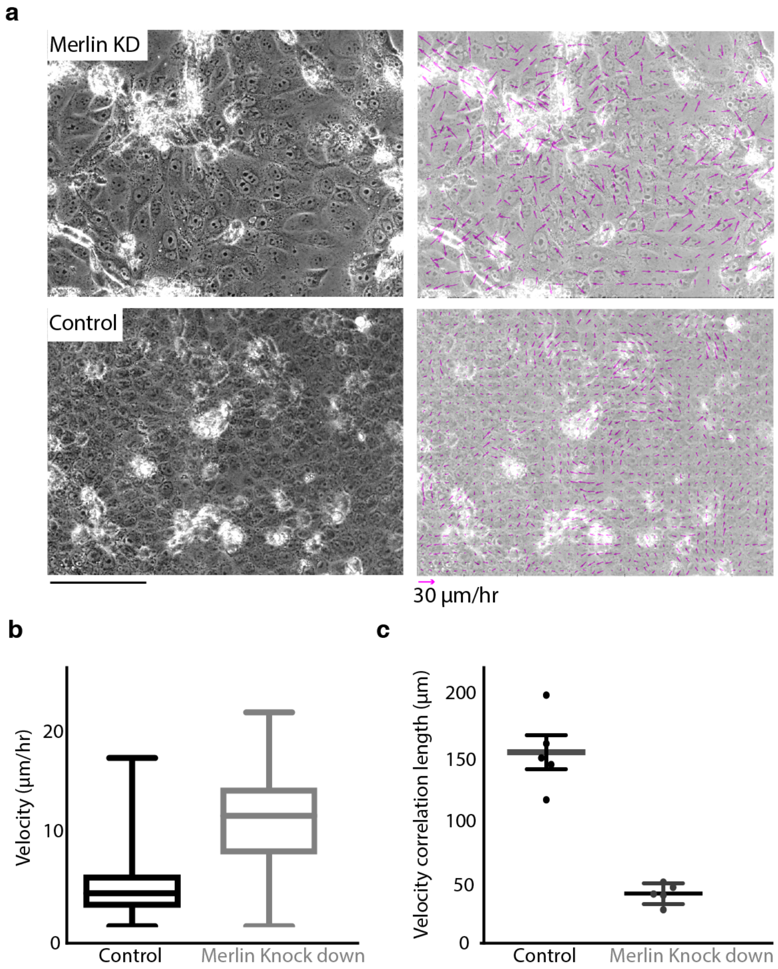

Supplementary Figure. 4. Merlin Knock down cells exhibit unjamming (a) Representative phase contrast images of merlin-depleted (Merlin KD) (*top panel*) and wild-type (Control) (*bottom panel*) cells, corresponding velocity profiles overlaid over phase contrast images showing a state of high fluidity or unjamming in merlin-depleted cells as compared to the control. **(b)** Box plot showing range of fluidity in control and merlin KD cells showing high fluidity on merlin KD cells. Data is pooled from three independent experiments, n = 3 **(c)** Scatter dot plot showing lower correlation of velocity in merlin KD cells as compared to control cells. Data is pooled from three independent experiments, n = 3, N= 5 where N is the number of data points. Whiskers in box plot show minimum to maximum range. Line in scatter dot plot display mean and error bars show S.E.M. Scale bars, 100 µm.

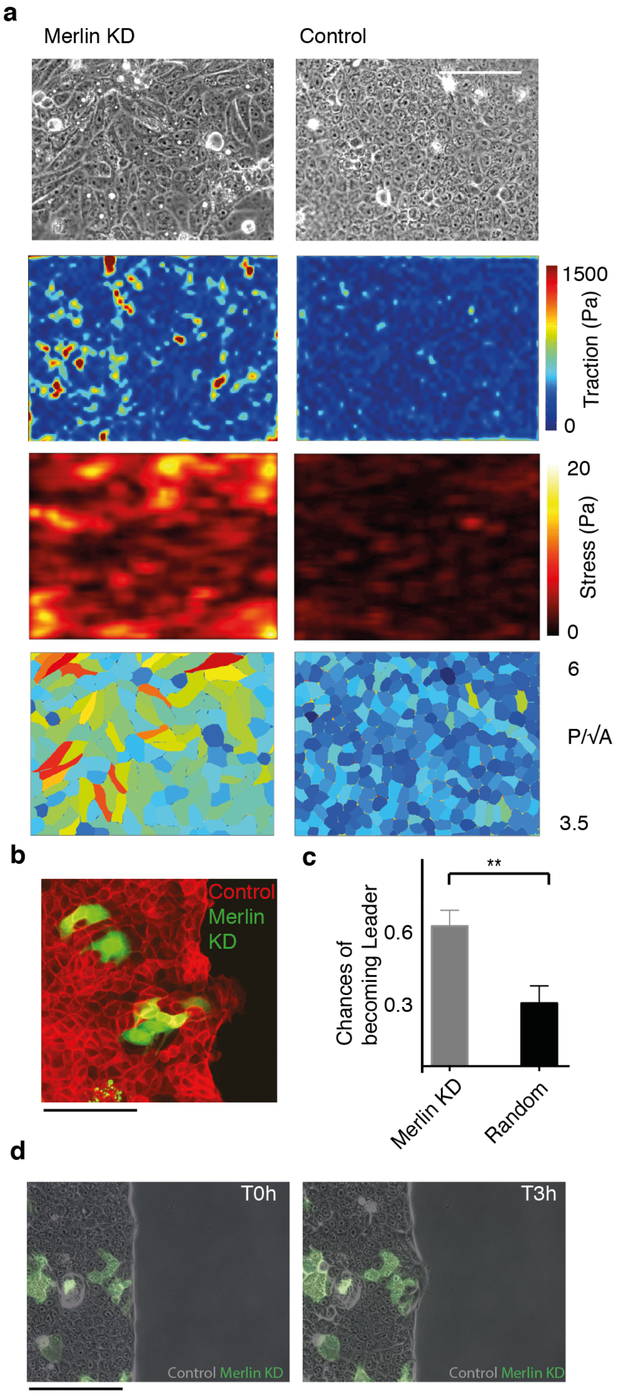

Supplementary Figure. 5. Introducing unjammed follower helps to stimulate leader cell formation. (a) Representative phase contrast images of merlin-depleted (Merlin KD) (*left*) and wild-type (Control) (*right*) cells, corresponding traction stress landscapes, average normal stress landscapes, and maps of the cellular shape indices (P/√A) showing a state of high fluidity or unjamming in merlin-depleted cells as compared to the control. **(b)** *LifeAct* MDCK cells (green) transfected with Merlin *siRNA1*, co-cultured with wild-type MDCK cells stained with phalloidin (red). Representative image show formation of leader cells triggered by unjammed follower behind them, Cells were fixed at T = 2h and stained for actin (red). **(c)** Bar graphs showing statistical distribution of chances of leader cell formation in front merlin-depleted, or random follower. **(d)** Time lapse imaging, *LifeAct* MDCK transfected with Merlin *siRNA2*, cocultured with wild type MDCK showing leader cell emerging from merlin depleted followers. Line represents mean and error bars represents S.D. Scale bars, 100 µm. Data collected from five independent experiments, n = 5. ** P<0.01, student's t-test.

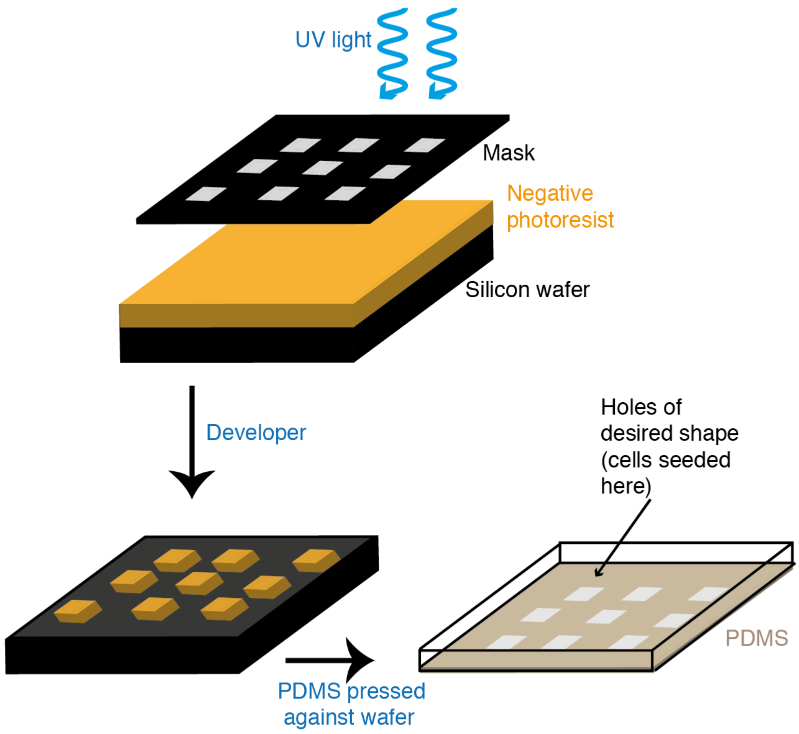

Supplementary Figure. 6. Production of PDMS micro-stencils. Schematic representation demonstrating fabrication of patterned micro-stencils. Black transparencies containing holes of desired shapes are used as masks to engrave about 50 µm thick structures of a negative photoresist onto a silicon wafer when illuminated with UV light (*top*). A developer removes the unexposed photoresist from the wafer but retains the cured structures (*bottom left*). PDMS pressed against the finished wafer at 65°C for 100 min produce holes of desired pattern in a thin PDMS membrane (*bottom right*).

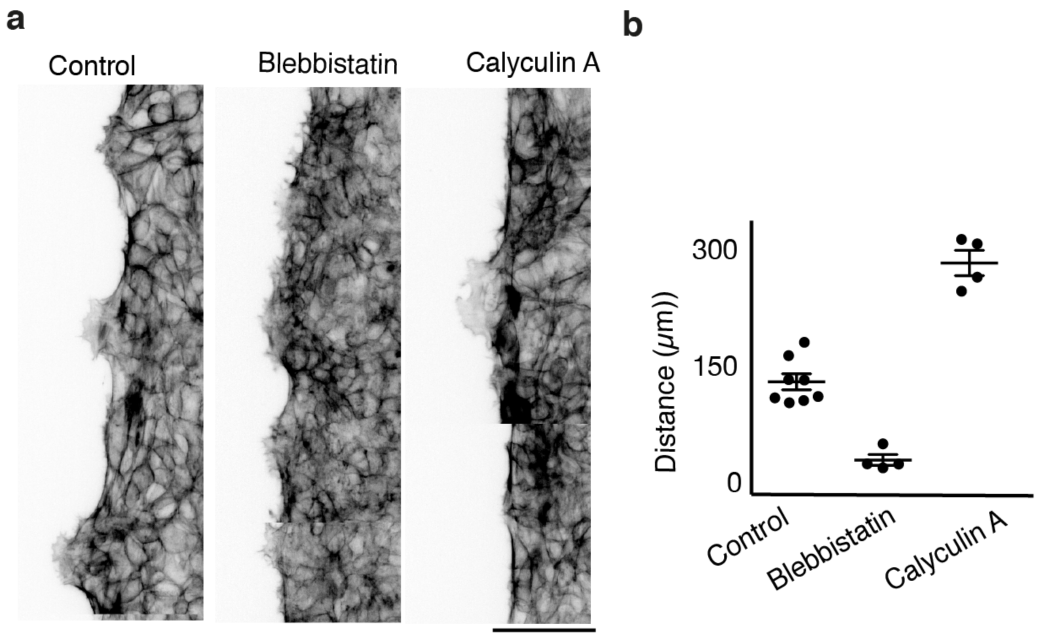

Supplementary Figure. 7. Modification of d_{LL} by drug treatments in HaCaT cells. (a) Representative actin staining images showing changing distance between leaders upon chemical modification in HaCaT cells in Control, blebbistatin and Calyculin-A (*left to right*) treated collectives cultured on glass coated with 50 µg/ml fibronectin. (b) Scattered dot plot showing distance between leaders in control, blebbistatin- and calyculin A-treated collectives. Line represents mean and error bars represents S.E.M. Scale bar, 100 µm. Data collected from three independent experiments, n = 3.

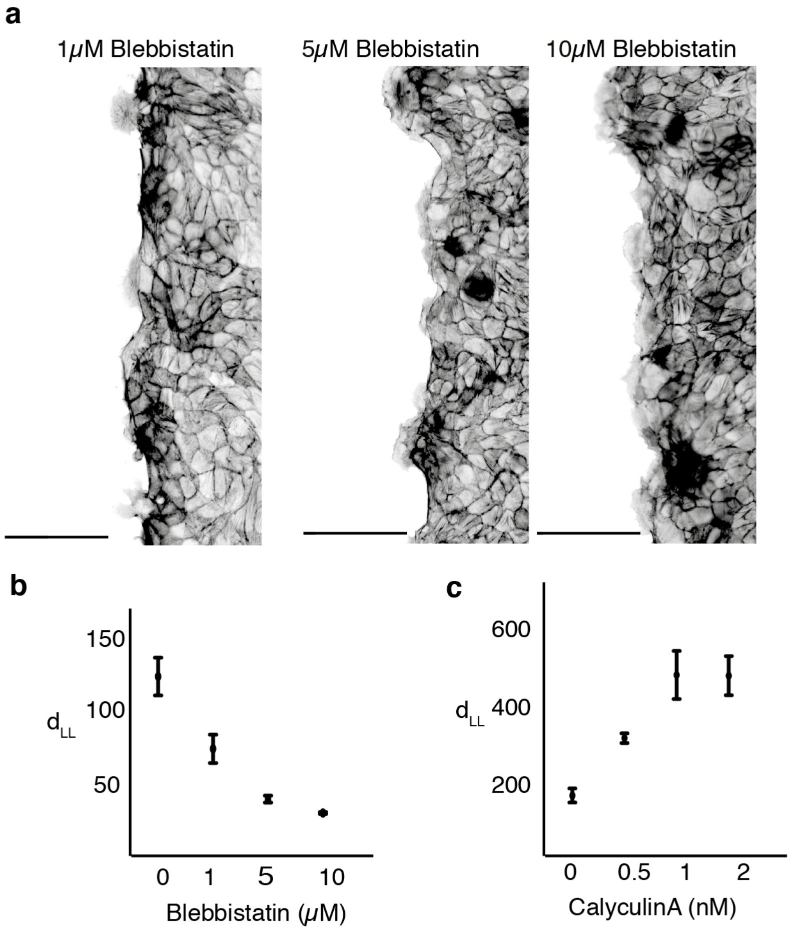

Supplementary Figure. 8. Modification of d_{LL} by using different concentrations of drugs. **(a)** Representative actin staining images showing decrease in distance between leaders upon increasing concentration of blebbistatin. **(b)** Statistics showing decreasing distance between leaders upon increasing blebbistatin concentrations. Data collected from three independent experiments, n = 3 and **(c)** increasing distance between leaders upon calyculin-A treatment. Data collected from four independent experiments, n = 4. Line represents mean and error bars represents S.E.M. Scale bar, 100 μm.

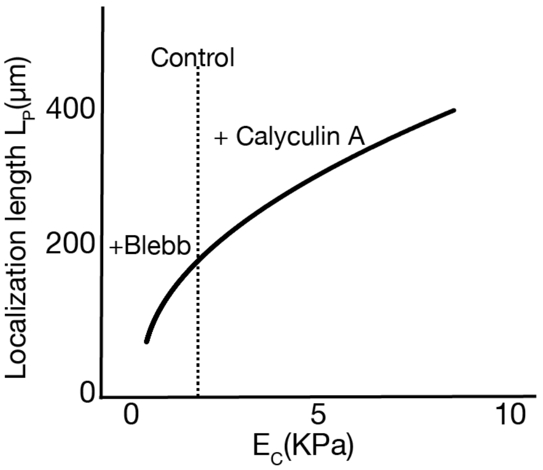

Supplementary Figure. 9. Localization length L_P as a function of the elastic modulus of the cell layer E_C as obtained from the theoretical continuum model

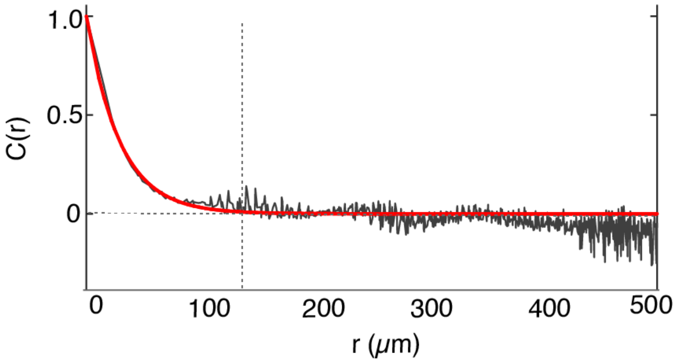

Supplementary Figure. 10. Representative correlation curve showing cell-cell forces are correlated.

**Quantity**	**Parameter**	**Value**
Elastic modulus of layer	E_C	700 Pa
Thickness of the layer	h_C	5 μm
Cell layer size	L	1000 μm
Length of subunit (i.e. cell)	l_{c0}	20 μm
Stiffness of focal adhesion bonds	k_a	2.5 nN/μm
Elastic modulus of the substrate	E_S	11KPa
Thickness of substrate	h_s	50 μm
Poisson's ratio of the substrate	v	0.5

Supplementary Table 1: **Cell and substrate parameters for the model**

**Cell group**	**E_C (KPa)**	**σ_0 (KPa)**	**F_L (nN)**
Control	0.7	1	100
Blebbistatin	0.1	0.5	10
Calyculin-A	5	5	400

Supplementary Table 2: Bulk parameters for control and drug treated cell monolayers
