# Supplementary material for: Mechanical interactions among followers determine the emergence of leaders in migrating epithelial cell collectives
Source: Nat Commun. 2018 Aug 27;9:3469. doi: 10.1038/s41467-018-05927-6 (PMC6110746; doi:10.1038/s41467-018-05927-6)
Supplement: Supplementary file 3 — Description of Additional Supplementary Files [file 41467_2018_5927_MOESM3_ESM.docx]

**Description of Additional Supplementary Files**

Supplementary Movie 1. **Biphasic behaviour in a migrating outgrowth.** Time lapse phase contrast imaging showing biphasic behaviour of the outgrowth monolayer in context of leader-cell formation. Phase-0 display no leader, Phase-1 is marked by leader cell at the tip of a migrating outgrowth and Phase-2 is marked by appearance of new leaders in the outgrowth and starts when extent of followers in the outgrowth exceed the length up-to which force from the leader cell can be transmitted.

Supplementary Movie 2. **Biphasic behaviour as seen in a scratch wound assay.** A confluent monolayer of LifeAct MDCK is scratched to observe the development of migrating outgrowths. Panels shows phase contrast (left) and GFP (right) channels. Outgrowths display similar biphasic behaviour as observed in confinement release wound healing assay.

Supplementary Movie 3. **Biphasic behaviour as seen in a scratch wound assay.** A confluent monolayer of LifeAct MDCK is scratched to observe the development of migrating outgrowths. Panels shows phase contrast (left) and GFP (right) channels. Outgrowths display similar biphasic behaviour as observed in confinement release wound healing assay.

Supplementary Movie 4. **Traction Force profile over time in the region where a leader cell is formed.** Transparent traction force profile overlaid on phase contrast images showing cellular tractions over time. Movie demonstrate increased traction force in followers leads to the leader cell formation at the edge.

Supplementary Movie 5. **Traction Force profile over time in the region where no leader cell is formed.** Transparent traction force profile overlaid on phase contrast images showing cellular tractions over time. Movie demonstrate lower traction force in followers in the regions behind nonleader as compared to the followers behind leaders (refer supplementary movie 4) 3

Supplementary Movie 6: **Monolayer stress profile over time in the region where a leader cell is formed.** Transparent monolayer stress profile overlaid on phase contrast images showing intercellular stress over time. Movie demonstrate increased cell-cell stress in followers leading to the leader cell formation at the edge.

Supplementary Movie 7. **Velocity profile over time.** Movie showing velocity profile over time of the migrating cellular monolayer.

Supplementary Movie 8. **Collective dynamics override interfacial bias.** Confinement release assay performed on LifeAct MDCK cultured in beak shaped micropatterns to bias leader cell formation at the edge (right panel) and unbiased micropattern (left panel). Time lapse imaging upon confinement removal show that distance between the leader cells in the end, is similar in both patterns showing collective dynamics overrides interfacial bias.

Supplementary Movie 9: **Modification of force correlation length modifies the distance between leaders.** Confinement release assay performed on monolayers treated with contractility inhibiting agent, blebbistatin (top right) and contractility enhancing agents, Calyculin A (bottom left) & RhoA activator, CN03 (bottom right)- Showing distance between the leaders is modified upon modifying cellular contractility. When contractility is low, leader cells can pull less and therefore more leaders are required (blebbistatin, top right). When contractility is high, leader cells can pull more and therefore less leaders are required (Calyculin A and RhoA activators, bottom panels)

Supplementary Movie 10: **Followers pull on the future leader to elect them to their fate.** LifeAct MDCK Cells cultured and allowed to migrate on a PDMS substrate with a micro-trench until they cross the trench. Unidirectional pulling from behind allow cells behind the trench to pull on the cells ahead of it. Imaging in phase contrast channel (left panel) and GFP chanel (right panel) show that when followers are forced to pull from behind (T 00:00), edge cells polarize and protrude like a leader (T 01:30)

Supplementary Movie 11: **Actin belt breaking is a consequence but is not sufficient for leader cell emergence.** An observation reporting not all the actin belt breakage leads to the emergence of a leader cell, showing importance of cellular dynamics in leader-cell formation:
